# Impacts of peat bulk density, ash deposition and rainwater chemistry on establishment of peatland mosses

**DOI:** 10.1007/s11104-017-3325-7

**Published:** 2017-07-05

**Authors:** Alice Noble, Sheila M. Palmer, David J. Glaves, Alistair Crowle, Joseph Holden

**Affiliations:** 1 water@leeds, School of Geography, University of Leeds, Leeds, UK; 2 Natural England, Foss House, Kings Pool, Peasholme Green, York, UK

**Keywords:** Burning, *Campylopus*, Fire, Invasive species, Nutrients, Pollution, *Sphagnum*

## Abstract

**Background and aims:**

Peatland moss communities play an important role in ecosystem function. Drivers such as fire and atmospheric pollution have the capacity to influence mosses via multiple pathways. Here, we investigate physical and chemical processes which may influence establishment and growth of three key moss species in peatlands.

**Methods:**

A controlled factorial experiment investigated the effects of different peat bulk density, ash deposition and rainwater chemistry treatments on the growth of *Sphagnum capillifolium*, *S. fallax* and *Campylopus introflexus*.

**Results:**

Higher peat bulk density limited growth of both *Sphagnum* species. *S. capillifolium* and *C. introflexus* responded positively to ash deposition. Less polluted rain limited growth of *C. introflexus*. Biomass was well correlated with percentage cover in all three species.

**Conclusions:**

Peat bulk density increases caused by fire or drainage can limit *Sphagnum* establishment and growth, potentially threatening peatland function. Ash inputs may have direct benefits for some *Sphagnum* species, but are also likely to increase competition from other bryophytes and vascular plants which may offset positive effects. Rainwater pollution may similarly increase competition to *Sphagnum*, and could enhance positive effects of ash addition on *C. introflexus* growth. Finally, cover can provide a useful approximation of biomass where destructive sampling is undesirable.

## Introduction

Peatlands are a globally important provider of ecosystem services including carbon storage, biodiversity and water quality maintenance. However, many peatlands have been degraded by human influences including atmospheric pollution and fire (Evans et al. 2014; Holden et al. 2007). In northern hemisphere peatlands *Sphagnum* spp. often play a key role in peat formation, so re-establishing or increasing *Sphagnum* cover is an important focus of restoration (Parry et al. 2014). A range of other bryophytes occur on peatlands, but dominance of less characteristic species at the expense of *Sphagnum* may threaten ecosystem function. One such species is *Campylopus introflexus*, an invasive species native to the southern hemisphere which has shown an expanded range and increased abundance on some northern hemisphere peatlands in recent decades (Equihua and Usher 1993).

Peatland vegetation is frequently influenced by multiple environmental drivers including grazing, cutting, prescribed burning, wildfire and atmospheric pollution (Holden et al. 2007). Each driver may influence vegetation via multiple pathways; alteration of the hydrological, thermal, chemical and biological properties of peat all have the potential to impact mosses (e.g. Bu et al. 2013; Lukenbach et al. 2015; Price and Whitehead 2001), potentially in conflicting ways. Knowledge of which processes exert the greatest control on important peatland moss species has the potential to inform the adaptation of management tools to support ecosystem function.

Fire is a common disturbance on peatlands globally and can take the form of wildfire or prescribed burning. Fire regimes are subject to change over time according to environmental conditions, human activity and policy. For example, Kasischke and Turetsky (2006) found that the incidence of fire increased across the North American boreal region from 1959 to 99. In the UK around 15% of land cover is peat and an estimated 18% (3150km^2^) of this has been subjected to prescribed burning (Worrall et al. 2010), mainly to manage vegetation for game or livestock. Douglas et al. (2015) observed an increase in the annual number of burns in the UK from 2001 to 2011. However, the environmental impacts of burning practices have been the subject of several studies and significant debate in recent years (Brown et al. 2016; Dougill et al. 2006). An important question in the debate is the effect of burning on *Sphagnum* spp., but past studies have produced conflicting conclusions, with *Sphagnum* sometimes increasing and sometimes declining after burning, and abundance varying with time since burn (Glaves et al. 2013). Increased knowledge of the pathways via which burning affects *Sphagnum* is therefore urgently required. *C.introflexus* has been observed to colonise rapidly after fire (Southon et al. 2012), including on many northern hemisphere peatlands where it is not native, but understanding of the processes controlling fire effects on mosses in general is still limited.

One way in which drivers including fire may influence mosses is by altering peat properties such as near-surface peat bulk density. Increased bulk density can reduce water availability to mosses, which are non-vascular and rely on passive capillary transport, but species may differ in their responses depending on water requirements and desiccation tolerance (Sagot and Rochefort 1996). Holden et al. (2014) measured a mean bulk density of 0.259 (±0.013) g cm^−3^ in peatland plots where the surface vegetation had been subjected to prescribed burning 2 years previously compared to 0.110 (±0.008) g cm^−3^ in unburned plots, potentially due to drying-induced compression and collapse of the peat mass after burning. Prescribed burns are normally controlled to burn vegetation without igniting the underlying peat. However, similar bulk density effect observed in a North American peatland was attributed to the exposure of denser peat when surface layers were consumed in a wildfire (Thompson and Waddington 2013). Near-surface bulk density may also be influenced by drivers including drainage (Ketcheson and Price 2011) and peat harvesting (Van Seters and Price 2002). While several studies have investigated the influence of water table on *Sphagnum* growth (Buttler et al. 1998; Grosvernier et al. 1997; Robroek et al. 2009), changes in bulk density have the potential to affect water availability to mosses even when the water-table depth is favourable by reducing soil water storage capacity, potentially increasing soil water retention, and slowing the rate at which water lost via evapotranspiration is replaced at the peat surface (Boelter 1968). These changes can reduce the amount of water loss needed to cause soil water pressure to fall below around −100 mb (Thompson and Waddington 2013), a point at which *Sphagnum* plants are unable to withdraw moisture from the peat (Price et al. 2003; Clymo and Hayward 1982).

Another process which may affect mosses after fire is ash deposition. Ash deposited after fire on peatlands is often largely a product of woody species such as heather (*Calluna vulgaris*). Nearly all nitrogen is lost during burning, but ash is a source of cations including Ca^2+^, Mg^2+^, Na^+^ and K^+^, which can impact soil water pH, as well as plant growth and competition (Allen 1964; Allen et al. 1969). At high concentrations these cations can have toxic effects, but at lower levels they can enhance growth of some species, particularly in ombrotrophic peatlands which may be nutrient limited (Hoosbeek et al. 2002; Vicherova et al. 2015). Ash may also block near-surface macropores in peat and alter hydrology, potentially affecting water availability to mosses (Holden et al. 2014). Much work on the effects of ash in peatland systems has focussed on the fertilisation of forest plantations with wood ash, which can cause visible damage to *Sphagnum* (Dynesius 2012). However, the application rate of ash for fertilisation can be around 20 times higher than the amount deposited after vegetation fire (Moilanen et al. 2002), and it is often applied in a processed form, so results from these studies may not be comparable to the effects of prescribed burning and wildfire.

In the absence of ash, rainwater is the most significant source of plant nutrients on ombrotrophic peatlands. Concentrations of nutrients including nitrogen and sulphur can vary regionally according to atmospheric pollution levels. Historically, *Sphagnum* abundance has been reduced in regions with high atmospheric pollution, including areas near industrial centres in the UK (Lee et al. 1987) where in the twentieth century *Sphagnum* previously dominant on peatlands was almost completely extirpated, leading to drying and erosion of peat (Carroll et al. 2009). Negative effects of sulphur have been documented in several *Sphagnum* species (Ferguson et al. 1978), but concentrations in rainwater have fallen in the UK in recent decades. Conversely, nitrogen levels have remained high in some regions (Carroll et al. 2009). On blanket peatlands nitrogen inputs are likely to benefit faster growing vascular plants, increasing competition to mosses (Malmer et al. 2003). *C introflexus* has been shown to respond positively to nitrogen inputs (Southon et al. 2012), but negative physiological effects on some *Sphagnum* species have been observed (Granath et al. 2012). It has been suggested that current levels of nitrogen and sulphur are not likely to prevent *Sphagnum* growth in the UK (Carroll et al. 2009), but individual species may respond differently. Furthermore, it is possible that rainwater chemistry interacts with ash deposition to influence nutrient dynamics.

In this study we use a controlled factorial experiment to isolate and quantify the effects of three variables related to fire and atmospheric pollution (peat bulk density, ash deposition and rainwater chemistry) on the establishment of *S. capillifolium*, *S. fallax* and *C. introflexus*. These species were chosen as they are common on blanket peatlands in the UK, and are thought to have varying preferences for moisture and nutrients (Table 1) which may influence their ability to establish on degraded peat. Based on existing knowledge of these species, we hypothesise that increased bulk density will affect *S. fallax* most negatively, followed by *S. capillifolium*, with little or no impact on *C. introflexus*. More polluted rainwater is expected to impact the two *Sphagnum* species, particularly *S. capillifolium*, negatively, but benefit *C. introflexus*. We would also expect ash to benefit *C. introflexus*, a species associated with burned areas, but to have a negative effect on both *Sphagnum* species, and for leached ash to have less impact than unleached ash.

**Table 1:** Ellenberg habitat indicator values for moisture and nitrogen (scale of 1–10, higher values indicate wetter conditions for moisture and more fertile conditions for nitrogen) for *S. capillifolium*, *S. fallax* and *C. introflexus,* adapted from Hill et al. (2007)

Species	*S. capillifolium*	*S. fallax*	*C. introflexus*
Moisture	Moist substrates (7)	Waterlogged substrates (9)	Moderately moist substrates (5)
Nitrogen	Extremely infertile sites (1)	Moderately infertile sites (3)	Infertile sites (2)

## Methods

### Substrate preparation

To collect peat as a substrate for the experiment with minimal disturbance to its structure, straight sided, bottomless pots were created using 6 cm lengths of 68 mm diameter PVC pipe. Peat was collected from an area of bare blanket peat, exposed by erosion, at Moor House-Upper Teesdale National Nature Reserve (henceforth Moor House) in the north of England. The site had not been burned for at least 60 years at the time of collection. For the pots with normal bulk density, 6 cm pots were pushed into the peat until level with the peat surface, taking care not to compress the peat, and removed. Pots with increased bulk density were prepared by inserting 10 cm lengths of pipe and then compressing the 10 cm peat core into 6 cm pots. Three sample pots from each treatment were dried to constant weight and mean bulk density was calculated to be 0.131 (±0.004) g cm^−3^ for normal pots and 0.169 (±0.030) g cm^−3^ for compressed pots. Pots were stored at 4 °C for two days prior to the start of the growth experiment.

### Rainwater preparation

Two types of artificial rainwater were produced to represent precipitation chemistry at UK upland sites with relatively low and high atmospheric pollution levels (Table 2). Chemical composition was based on ECN data (Rennie et al. 2015) averaged over 5 years (2007–2012) for two sites: Allt a’Mharcaidh in the Cairngorms, Scotland (less polluted) and Wardlow Hay Cop in the Peak District, England (more polluted). Concentrated solutions were made every four weeks by dissolving compounds (NaCl, MgSO_4_, CaSO_4_.2H_2_O and NH_4_NO_3_) in a 250 mL volumetric flask of deionised water at 100 times the required strength. The concentrated solutions were stored at 4 °C in 50 mL plastic vials and diluted with deionised water as required. HCl (0.1 M) was then added dropwise as necessary to give a pH between 5.2 and 5.8 for both rainwater types.

**Table 2:** Composition of the two artificial rainwater types produced for the experiment

Component	Cairngorm water meq L^−1^	Peak District water meq L^−1^
Ca^2+^	0.002	0.022
Cl-	0.075	0.073
Mg^2+^	0.016	0.015
Na+	0.074	0.067
NH_4_^+^	0.011	0.028
NO_3_^−^	0.011	0.028
SO_4_^2−^	0.018	0.036

### Ash preparation

To make the ash, vegetation consisting almost entirely of *C. vulgaris* was harvested at Moor House by cutting stems close to the ground. In the lab, vegetation was cut into 5 cm lengths, oven dried for 24 h at 105 °C, weighed, and then ignited in a muffle furnace at 450 °C. This temperature was chosen as it is comparable to temperatures recorded during prescribed burning in the field (Whittaker 1961). One third of the 108 pots used in the experiment had no ash added, while ash was scattered on the surface of the remaining two thirds at a rate of 10 g m^−2^ to correspond to a dry weight of 672 g m^−2^, the average *C.vulgaris* biomass 16 years after burning recorded by Alday et al. (2015) at Moor House. Half of the ash added was subject to leaching with artificial rainwater (corresponding with the type to be supplied during the experiment) before adding to pots at a rate of 413 L m^−2^ (1.5 L per pot), which approximates a mean value for total spring (March, April and May) rainfall at Moor House (Rennie et al. 2015). In the UK, burning is restricted to the winter half year (October–April), so this amount is comparable to the rain a site might receive between burning and the growing season. For the growth experiment (section 2.4), ash was leached on filter paper rather than in situ on the pots as this was quicker and avoided confounding effects of the leaching process (e.g. differential drying of leached and unleached pots) on moss growth. However, for the chemical impacts on peat experiment (section 2.5), ash was added to the peat surface before the start of the leaching process.

### Growth experiment

Bryophyte material was collected from Moor House (*S. capillifolium* and *S. fallax*) and Leek Moors in the Peak District (*C. introflexus*). Diaspores comprising individual capitula for both *Sphagnum* species and individual stems for *C. introflexus* were prepared and ten diaspores of an individual species were added to each pot. Diaspores were initially watered in from above with 50 mL of artificial rainwater, equivalent to 13.77 mm or around 3 days spring rainfall at Moor House (Rennie et al. 2015), per pot. Pots were arranged in 18 plastic trays, each of which contained six pots representing the six bulk density-species combinations. Ash and rainwater treatments were the same within individual trays to avoid nutrient contamination. Each species x bulk density x ash x rainwater combination was replicated three times, giving 108 pots in total.

The growth experiment took place in a controlled environment room with two LED light units (Heliospectra AB, Sweden), which were on between 5 am and 9 pm to simulate spring/summer daylight hours in the UK. The temperature in the environment room was 9 °C at night (9 pm to 9 am) and 14 °C during the day. These temperatures are comparable to mean summer temperatures at UK upland sites (Rennie et al. 2015).

The experiment was run for 152 days, during which the two rainwater types were supplied to pots by filling the trays as required to maintain a water-table depth between 2 cm and 5 cm. The following variables were recorded for each pot every 14 days: survival (proportion of diaspores which had not dried out), proportion of diaspores which had produced new material, cover (percentage of the peat surface covered by moss) and maximum height of moss. At the end of the experiment, all moss was harvested from the peat surface of each pot using tweezers, air dried to constant weight, and the final dry biomass was recorded as a measure of growth.

### Chemical impacts of ash addition and leaching

Separately from the growth experiment, the impact of ash addition and leaching on peat chemistry was investigated using 18 samples. At the start of the leaching process, 18 pots of peat were wetted with 250 mL of artificial rainwater and allowed to drain. Ash was then added to half of the pots as described in section 2.3. Twelve of the cores were subjected to leaching at the rate described in section 2.3; six with each rainwater type. Water was added to all pots at the rate it drained from the slowest draining pots; approximately 200 mL a day, and pots were covered with plastic sheeting to reduce evaporation.

After the leaching process, surface peat samples were taken from all 18 pots and BaCl_2_ extractions were performed using 5 g of fresh peat and 45 mL of 0.1 M BaCl_2_ per sample. The resulting mixture was shaken for 2 h at 15 rpm, centrifuged for 15 min at 2115 rpm and filtered through Whatman No 41 filter paper before analysis of exchangeable Ca^2+^, Mg^2+^, Na^+^ and K^+^ by ICP-OES. Concentrations measured were converted from mg L^−1^ to cmol_c_ kg^−1^ dry weight for reporting.

### Data analysis

All statistical analyses were carried out using R 3.2.3 (R Development Core Team 2010) with the packages car (Fox and Weisberg 2011), nlme (Pinheiro et al. 2016), lsmeans (Lenth 2016) and ggplot2 (Wickham 2009).

Correlations between the different measurements of moss establishment (biomass and final measurements of survival, height and cover) were assessed using Pearson’s product-moment correlation. Mixed ANOVA, accounting for the split-plot experimental design, was used to test effects of bulk density, ash and rainwater type and their interactions on dry biomass of each species separately. The Tukey HSD test was used to test for pairwise differences. Normality of residuals and homogeneity of variables were inspected graphically for all models.

Natural logarithm transformation was applied to the cation concentration data from the peat samples to reduce heteroscedasticity. Factorial ANOVA and Tukey HSD tests were then carried out to investigate the effects of ash and rainwater type on concentrations of Ca^2+^, Mg^2+^, Na^+^ and K^+^ in peat samples.

## Results

### Moss biomass

Air dried moss biomass was used as the dependent variable in the analyses as it represented the most objective measure of establishment success and was strongly correlated with final measurements of cover, survival, growth and height in both *Sphagnum* species (Table 3). Survival of the original diaspores of *C. introflexus* was uniformly low, as was height in this species, but biomass was well correlated with cover and the proportion of diaspores which gave rise to new growth.

**Table 3:** Pearson’s product moment correlation of biomass (g dry weight) with proportion of pot covered by moss, number of moss fragments surviving, number of fragments showing signs of growth and moss height. *P* values for all correlations shown are <0.001. N/A = not tested

Species	Cover %	Survival proportion	Growth proportion	Height (mm)
*Sphagnum capillifolium*	0.78	0.66	0.70	0.66
*Sphagnum fallax*	0.84	0.68	0.68	0.70
*Campylopus introflexus*	0.82	N/A	0.51	N/A

**Fig. 1:**
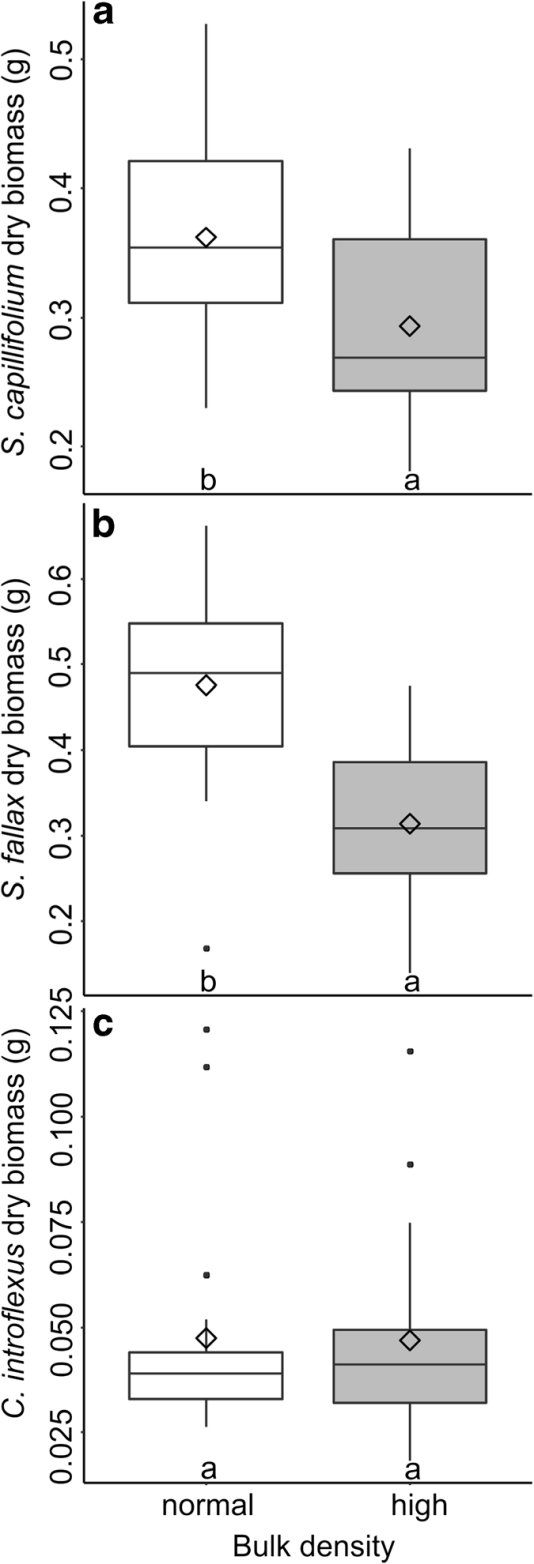
Boxplot showing dry biomass of a) *S. capillifolium* and b) *S. fallax* grown in pots with normal or high peat bulk density (*n* = 54). The horizontal line, box, whiskers, dots and ◊ indicate the median, upper and lower quartiles, minimum and maximum excluding outliers, outliers and mean respectively. Treatments which do not share a letter are significantly different (*p* < 0.05)

**Fig. 2:**
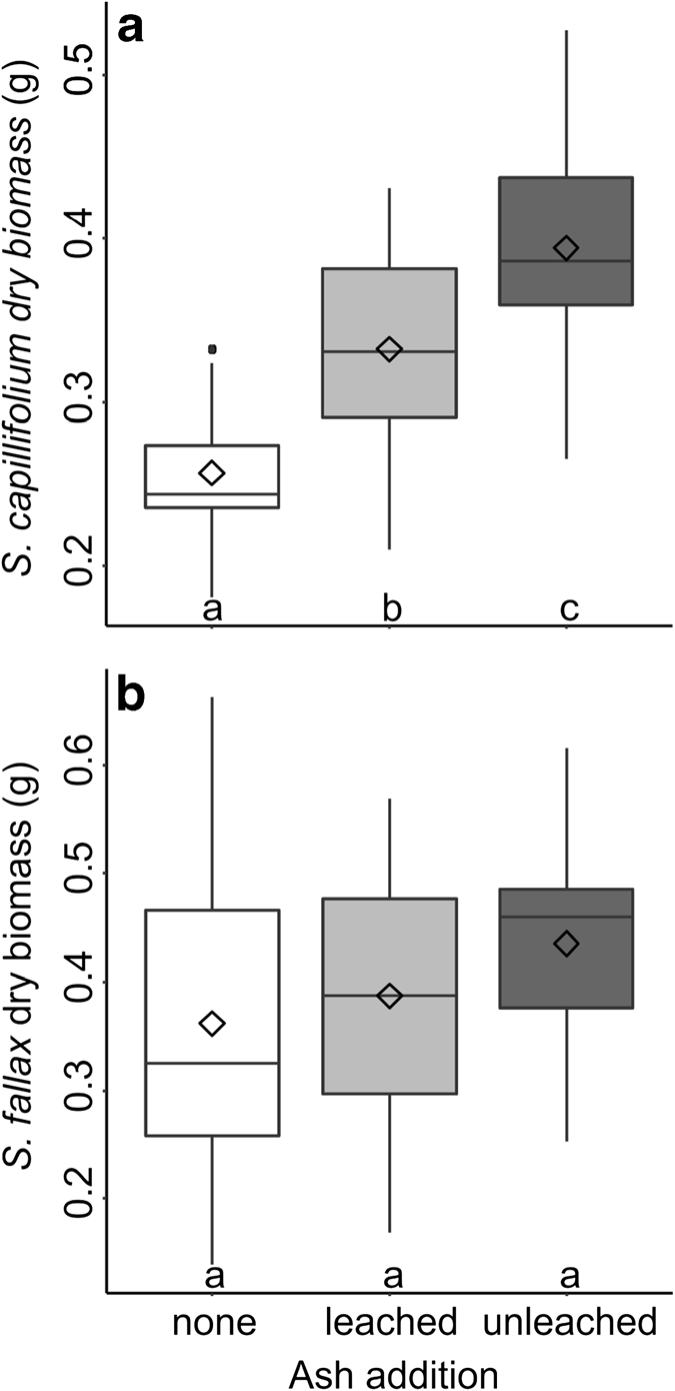
Boxplot showing dry biomass of *S. capillifolium* grown in pots with no ash, leached ash or unleached ash (*n* = 36). The horizontal line, box, whiskers, dots and ◊ indicate the median, upper and lower quartiles, minimum and maximum excluding outliers, outliers and mean respectively. Treatments which do not share a letter are significantly different (*p* < 0.05)

ANOVAs carried out on the biomass data indicated that the effects of the three treatments manipulated in the experiment varied depending on moss species (Table 4). Pots with normal bulk density produced greater biomass of both *S. capillifolium* (*F* = 16.55, *p* = 0.002) and *S. fallax* (*F* = 18.33, *p* = 0.001) compared to those with high peat bulk density (Fig. 1). There was no significant effect of bulk density on *C. introflexus* (*F* < 0.01, *p* = 0.950). Ash addition impacted *S. capillifolium* (*F* = 15.28, *p* = 0.001) and Tukey HSD tests indicated significantly higher biomass in pots with unleached ash than those with leached ash or no ash, and likewise in pots with leached ash compared to those with no ash (Fig. 2). In *S. fallax*, no significant impact of ash on biomass was observed (*F* = 1.76, *p* = 0.213).

**Table 4:** Results of mixed ANOVA of final biomass (g dry weight) of *S. capillifolium*, *S. fallax* and *C. introflexus* according to water chemistry, ash treatment and peat bulk density

Species	Variable	Degrees of freedom	Sum of Squares	Mean Square	F value	Pr(>F)
S. capillifolium	Ash	2	0.113	0.057	15.281	0.001
	Rainwater	1	0.006	0.006	1.513	0.242
	Ash:rainwater	2	0.006	0.003	0.805	0.470
	Residuals	12	0.044	0.004		
	Bulk density	1	0.042	0.042	16.551	0.002
	Ash:bulk	2	0.002	0.001	0.456	0.644
	Rainwater:bulk	1	0.001	0.001	0.328	0.577
	Ash:rainwater:bulk	2	0.003	0.002	0.643	0.543
	Residuals	12	0.031	0.003		
S. fallax	Ash	2	0.033	0.017	1.764	0.213
	Rainwater	1	0.007	0.007	0.708	0.417
	Ash:rainwater	2	0.015	0.008	0.800	0.472
	Residuals	12	0.113	0.009		
	Bulk density	1	0.237	0.237	18.334	0.001
	Ash:bulk	2	0.035	0.018	1.374	0.290
	Rainwater:bulk	1	0.002	0.002	0.189	0.671
	Ash:rainwater:bulk	2	0.002	0.001	0.065	0.937
	Residuals	12	0.155	0.013		
C. introflexus	Ash	2	0.005	0.003	16.610	<0.001
	Rainwater	1	0.003	0.003	20.670	0.001
	Ash:rainwater	2	0.003	0.002	10.260	0.003
	Residuals	12	0.002	0.000		
	Bulk density	1	0.000	0.000	0.004	0.950
	Ash:bulk	2	0.000	0.000	0.160	0.854
	Rainwater:bulk	1	0.000	0.000	0.010	0.921
	Ash:rainwater:bulk	2	0.000	0.000	0.037	0.964
	Residuals	12	0.008	0.001

**Fig. 3:**
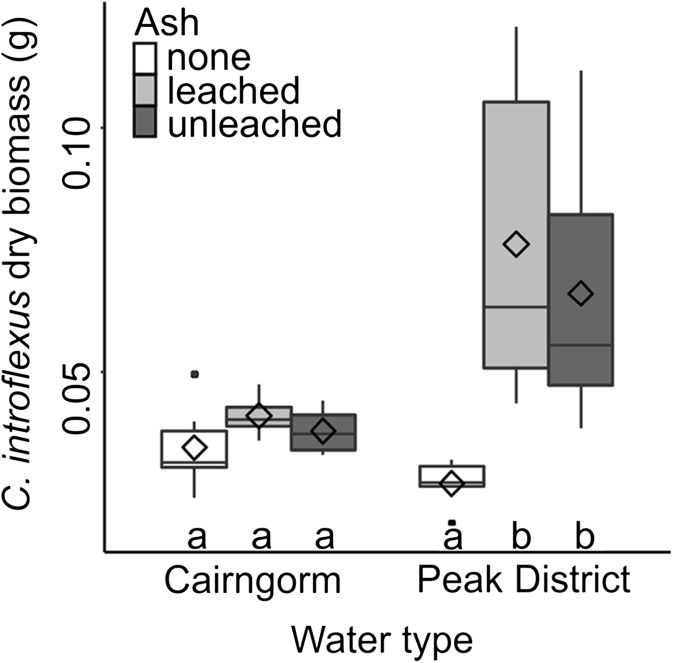
Boxplot showing dry biomass of *C. introflexus* grown in pots with no ash, leached ash or unleached ash and Cairngorm or Peak District water chemistry (*n* = 18). The horizontal line, box, whiskers, dots and ◊ indicate the median, upper and lower quartiles, minimum and maximum excluding outliers, outliers and mean respectively. Treatments which do not share a letter are significantly different (*p* < 0.05)

Ash (*F* = 16.61, *p* < 0.001), rainwater chemistry (*F* = 20.67, *p* = 0.001) and the interaction between the two (*F* = 10.26, *p* = 0.003) impacted *C. introflexus* biomass. Pots watered with Peak District rainwater overall produced higher *C. introflexus* biomass than those watered with Cairngorm rainwater. The impact of ash was nonsignificant in pots watered with Cairngorm water, but pots watered with Peak District rainwater had lower biomass when no ash was added compared to unleached and leached ash (Fig. 3). Rainwater chemistry did not significantly impact biomass of either *S. capillifolium* (*F* = 1.51, *p* = 0.242) or *S. fallax* (*F* = 0.71, *p* = 0.417).

### Impacts of ash addition and leaching on peat chemistry

**Fig. 4:**
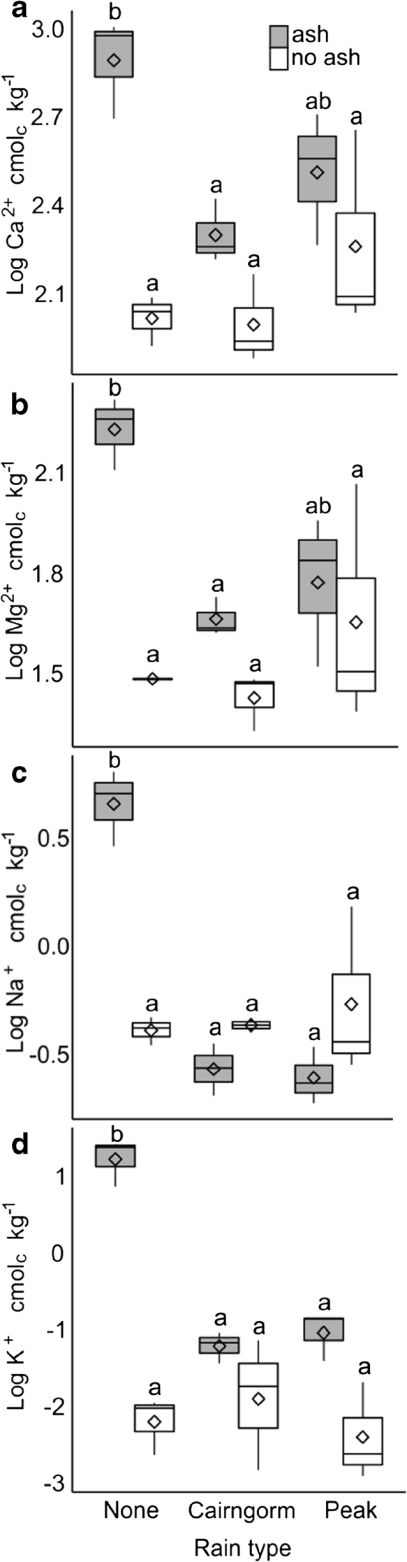
Concentrations (natural log cmol_c_ kg^−1^ dry weight) of Ca^2+^, Mg^2+^, Na^+^ and K^+^ in surface peat samples taken from pots with and without ash additions and either left unleached or leached with 1.5 L of either Cairngorm or Peak District recipe artificial rainwater (*n* = 3). The horizontal line, box, whiskers, dots and ◊ indicate the median, upper and lower quartiles, minimum and maximum excluding outliers, outliers and mean respectively. Treatments which do not share a letter are significantly different (*p* < 0.05)

Factorial ANOVA and Tukey HSD tests revealed significantly greater concentrations of Ca^2+^, Mg^2+^, Na^+^ and K^+^ in unleached peat samples with ash added compared to unleached samples without ash (*p* = 0.002; 0.004; <0.001; <0.001 respectively). However, differences in the concentrations of Ca^2+^, Mg^2+^, Na^+^ and K^+^ between samples with and without ash were not significant after leaching with either Cairngorm (*p* = 0.465; 0.634; 0.786; 0.574) or Peak District (*p* = 0.650; 0.964; 0.327; 0.054) rainwater (Fig. 4).

## Discussion

The results of this study showed that growth of all three moss species responded differently to the experimental treatments. Both *Sphagnum* species were affected by bulk density and *S. capillifolium* was affected by ash addition, whilst *C. introflexus* was affected by both ash addition and rainwater chemistry. This highlights the importance of considering species individually and indicates that even species with a similar functional niche may respond differently to environmental change. Of the variables measured, cover was best correlated with biomass in all three species (Table 3), suggesting that recording percentage cover would be an appropriate alternative to biomass measurements in experiments where destructive sampling is not possible. Comparison of mean values for biomass and cover indicate that the differences in biomass observed within treatments represented ecologically significant differences in percentage cover (Table 5).

**Table 5:** Mean and standard error values for final biomass (g dry weight) and percentage cover of *S. capillifolium*, *S. fallax* and *C. introflexus* according to water chemistry, ash treatment and peat bulk density

		*Sphagnum capillifolium*		*Sphagnum fallax*		*Campylopus introflexus*	
Factor	Level	Biomass (g)	Cover %	Biomass (g)	Cover %	Biomass (g)	Cover %
Bulk density	Normal	0.36 ± 0.08	58 ± 14	0.48 ± 0.12	57 ± 18	0.05 ± 0.03	10 ± 6
	High	0.29 ± 0.07	42 ± 17	0.31 ± 0.09	41 ± 14	0.05 ± 0.02	14 ± 10
Ash addition	None	0.26 ± 0.05	34 ± 14	0.36 ± 0.16	44 ± 19	0.03 ± 0.01	2.2 ± 1.9
	Leached	0.33 ± 0.07	51 ± 12	0.39 ± 0.13	44 ± 17	0.06 ± 0.03	17 ± 7
	Unleached	0.39 ± 0.07	65 ± 9	0.44 ± 0.10	59 ± 15	0.05 ± 0.02	16 ± 5
Rainwater type	Cairngorm	0.34 ± 0.10	50 ± 18	0.38 ± 0.11	48 ± 15	0.04 ± 0.01	10 ± 6
	Peak	0.32 ± 0.07	50 ± 17	0.41 ± 0.15	50 ± 21	0.06 ± 0.03	14 ± 10

The negative impact of increased bulk density on both *S. capillifolium* and *S. fallax* suggests that water availability to these species was affected at higher bulk density. This could be the result of reduced water storage capacity, increased water retention, and decreased hydraulic conductivity associated with higher bulk densities. These conditions can increase the incidence of low soil water pressures (e.g. below -100mb), which would be unsuitable for *Sphagnum* (Price 1997; Clymo and Hayward 1982). Based on data provided by Boelter (1968) and Thompson and Waddington (2013) the difference in bulk density between the normal pots and the compacted pots may equate to a difference in volumetric water content between these treatments of ~0.1 at -100mb. Although water tables in the experiment were within a range considered favourable to *Sphagnum* (2 to 5 cm), the surfaces of many of the high bulk density pots were visibly drier than those with normal bulk density throughout the experiment, suggesting that water was less able to move through the peat matrix to replace evaporative loss at the surface. Clymo and Hayward (1982) identified this as a key potential issue for *Sphagnum*, even when there are relatively shallow water table conditions. In future work, measuring water pressure and/or water content at the peat surface could help to quantify this effect and develop our understanding of critical thresholds for *Sphagnum*. The fact that *C. introflexus* was unaffected reflects the species’ lower Ellenberg indicator value for moisture (Table 1) and could indicate a risk of a shift to species which can tolerate lower water availability at high bulk density. Holden et al. (2014) found that peat bulk densities decreased with time since burning, so leaving sites unburned for longer may create more favourable conditions for *Sphagnum* establishment. However, the high bulk density treatment in this experiment was actually lower than many of the bulk densities measured on burned plots by Holden et al. (2014), suggesting that greater limitations to *Sphagnum* establishment may occur in the field. On the other hand, the bulk density - water retention plots presented by Thompson and Waddington (2013) for soil water pressures of -100mb flatten out considerably for bulk densities >0.17 g cm-3 and so the effects on water retention, at -100mb, of enhanced bulk density beyond the range we tested may not be substantially greater in the field for fire affected sites. The normal bulk density treatment was similar to bulk densities measured by Holden et al. (2014) on unburned plots. As water was supplied from below throughout the experiment, water intercepted from precipitation was not accounted for, but past work has shown that dependence on precipitation can increase susceptibility to drought and reduce carbon uptake in *Sphagnum* (Nijp et al. 2014).

The positive impact of ash on *S. capillifolium* was unexpected given previous reports of damage to *Sphagnum* by ash (Dynesius 2012). This may be due to inputs of limiting nutrients since pots with unleached ash produced greater *S. capillifolium* biomass than pots with leached ash. Although concentrations of Ca^2+^, Mg^2+^, Na^+^ and K^+^ in peat with leached ash were not significantly different to those without ash, it is possible that small residual quantities of these or other nutrients had an effect, or that another nutrient was less easily leached. An increase in pH as a result of ash addition could also have affected *Sphagnum* growth. *Sphagnum* usually prefers acidic habitats (Clymo 1963), but tolerance ranges and optima can vary between species (Haraguchi 1996). The fact that rainwater chemistry had no impact on either *Sphagnum* species may suggest that the effect was down to a nutrient supplied by the ash and lacking in the rainwater. Phosphorus has been observed to affect *Sphagnum* both positively and negatively depending on the species and concentrations of other nutrients (Carfrae et al. 2007; Li et al. 1993; Sottocornola et al. 2007), but was found by Allen (1964) to leach from ash at a similar rate to magnesium. It is possible that ash addition from burning may facilitate establishment of *S. capillifolium* under some circumstances, but the benefit may be decreased if burning is shortly followed by a heavy rainfall event that leaches deposited ash. Furthermore, positive effects may be offset by the negative impacts of increased bulk density as well as increased competition from vascular plants and other mosses (including *C. introflexus*) benefiting from ash inputs. The fact that a similar pattern was not observed in *S. fallax* indicates that *Sphagnum* species vary in their responses to ash addition. Work by Brown et al. (2014) suggests that repeated prescribed burning may reduce peat cation exchange capacity, so in the field initial cation enrichment may be followed by depletion in the longer term.

*C. introflexus* was the only species which showed a response to rainwater chemistry, which is consistent with reports of a positive response to nitrogen increases (Southon et al. 2012). The interaction of rainwater chemistry with ash addition suggests that *C. introflexus* is limited by more than one nutrient, as biomass was greatest under nutrient inputs from both ash and Peak District rain. Neither of the *Sphagnum* species showed a response to rainwater chemistry, which suggests that current atmospheric pollution deposition is not directly limiting to these species. However, there is potential for competitive effects from other species including *C. introflexus* in the field. Given that the Ellenberg value for *S. fallax* indicated a higher nitrogen tolerance than *C. introflexus* (Table 1), a positive response to increased rainwater nutrients may have been expected. However, previous experimental work has shown a negative physiological response of the species to nitrogen increases, and it is suggested that local environmental conditions and supply of other nutrients may play a role in determining net impacts (Granath et al. 2012).

Overall, the results of this study show that anthropogenic influences including fire and atmospheric pollution can impact mosses via both physical and chemical processes. Effects were not consistent across the three species studied and in the case of *S. capillifolium*, ash addition and bulk density increase had opposing effects. The positive effect of ash addition on *S. capillifolium* may indicate that in some instances, ash can facilitate colonisation. However, increased competition from other mosses and vascular plants is likely to be a significant factor in the field. The negative impact of increased bulk density on both *Sphagnum* species indicates that peat physical properties governing water availability are an important factor to consider alongside water table depth in restoration efforts. Species specific responses highlight the importance of differentiating between moss species to ensure appropriate management and considering both species identity and local environmental conditions when planning *Sphagnum* re-introductions.
